# Case Report: Pancreatic amphicrine-like carcinoma with acinar differentiation harboring a KANK4-RAF1 gene fusion

**DOI:** 10.3389/pore.2026.1612537

**Published:** 2026-08-11

**Authors:** Sabrina Kammerer, Peter Bode, Heide Schreiber, Guacimara Ortega Sanchez

**Affiliations:** 1 Department of Medical Oncology and Hematology, Kantonsspital Winterthur, Switzerland; 2 Department of Pathology, Kantonsspital Winterthur, Switzerland

**Keywords:** acinar differentiation, exocrine and endocrine differentiation, KANK4::RAF1, metastatic, pancreatic amphicrine-like carcinoma

## Abstract

Pancreatic amphicrine-like carcinoma (ALC) is an exceptionally rare neoplasm characterized by simultaneous exocrine and endocrine differentiation within the same tumour cells. These tumours represent a diagnostic challenge because they must be distinguished from mixed neuroendocrine–non-neuroendocrine neoplasms (MiNENs), which consist of morphologically distinct tumour components. We report a case of pancreatic ALC with acinar differentiation harboring a KANK4::RAF1 fusion identified by comprehensive genomic profiling. Histologically, the tumour demonstrated acinar differentiation with expression of trypsin and BCL10 together with neuroendocrine differentiation characterized by synaptophysin and INSM1 expression within the same neoplastic population. Molecular analysis revealed a RAF1 rearrangement, a potentially actionable alteration previously described in a subset of pancreatic acinar carcinomas. The patient showed rapid disease progression despite systemic chemotherapy. Treatment with the MEK inhibitor trametinib was initiated based on the presence of a RAF1 fusion but was discontinued after 1 month because of toxicity, preventing assessment of therapeutic efficacy. This case expands the molecular spectrum of pancreatic ALC with acinar differentiation and highlights the importance of comprehensive molecular profiling in rare pancreatic neoplasms to identify potentially actionable genomic alterations.

## Introduction

Pancreatic epithelial neoplasms comprise a heterogeneous group of tumours with distinct morphological, immunophenotypic, and molecular characteristics. Although pancreatic ductal adenocarcinoma accounts for approximately 90% of malignant pancreatic tumors, several uncommon entities—including acinar cell carcinoma (ACC), pancreatic neuroendocrine tumors, pancreatoblastoma, and solid pseudopapillary neoplasms—display unique biological behavior and require different diagnostic and therapeutic approaches [1–3].

Amphicrine-like carcinoma (ALC) of the pancreas is an exceptionally rare neoplasm characterized by the coexistence of exocrine and endocrine differentiation within the same neoplastic cells. This distinguishes it from mixed neuroendocrine–non-neuroendocrine neoplasms (MiNENs), in which the two components are morphologically distinct and represent separate tumour populations. The current WHO Classification of Tumours (6th edition) recognizes amphicrine differentiation as a distinct pathological phenomenon, emphasizing the importance of integrating morphology with immunohistochemical findings to establish the diagnosis [4].

Recent advances in comprehensive genomic profiling have expanded the understanding of the molecular landscape of pancreatic acinar neoplasms. Recurrent alterations involving the MAPK signaling pathway, including RAF1 gene rearrangements, have been identified in a subset of acinar carcinomas, providing insight into tumour biology and raising the possibility of targeted therapeutic strategies. However, the spectrum of RAF1 fusion partners remains incompletely characterized, particularly in tumours exhibiting amphicrine differentiation [5, 6].

Here, we describe a rare case of pancreatic ALC with acinar differentiation harboring a KANK4-RAF1 fusion. To our knowledge, this specific fusion has not previously been reported in this tumour type. This case highlights the diagnostic challenges posed by amphicrine pancreatic neoplasms and underscores the value of comprehensive molecular profiling for identifying potentially actionable genomic alterations in rare pancreatic tumours.

## Case report

A 52-year-old woman was referred to our hospital with several months of persistent upper abdominal pain, bloating, and unintentional weight loss. Her symptoms had initially improved with pantoprazole therapy but subsequently worsened over time.

Laboratory investigations showed anaemia, with reduced haemoglobin (116 g/L), mean corpuscular volume (62 fL), and mean corpuscular haemoglobin (21 pg), while the mean corpuscular haemoglobin concentration remained within the normal range (336 g/dL). Liver enzymes were elevated, including lactate dehydrogenase (LDH 233 U/L), aspartate aminotransferase (AST 57 U/L), alanine aminotransferase (ALT 96 U/L), gamma-glutamyltransferase (GGT 235 U/L), and alkaline phosphatase (ALP 151 U/L). Total bilirubin was within normal limits (9.1 μmol/L). Tumour markers were largely unremarkable, with only a mild elevation in neuron-specific enolase (15.2 μg/L), while CA 19-9, CEA, and chromogranin A were within normal ranges.

**FIGURE 1:**
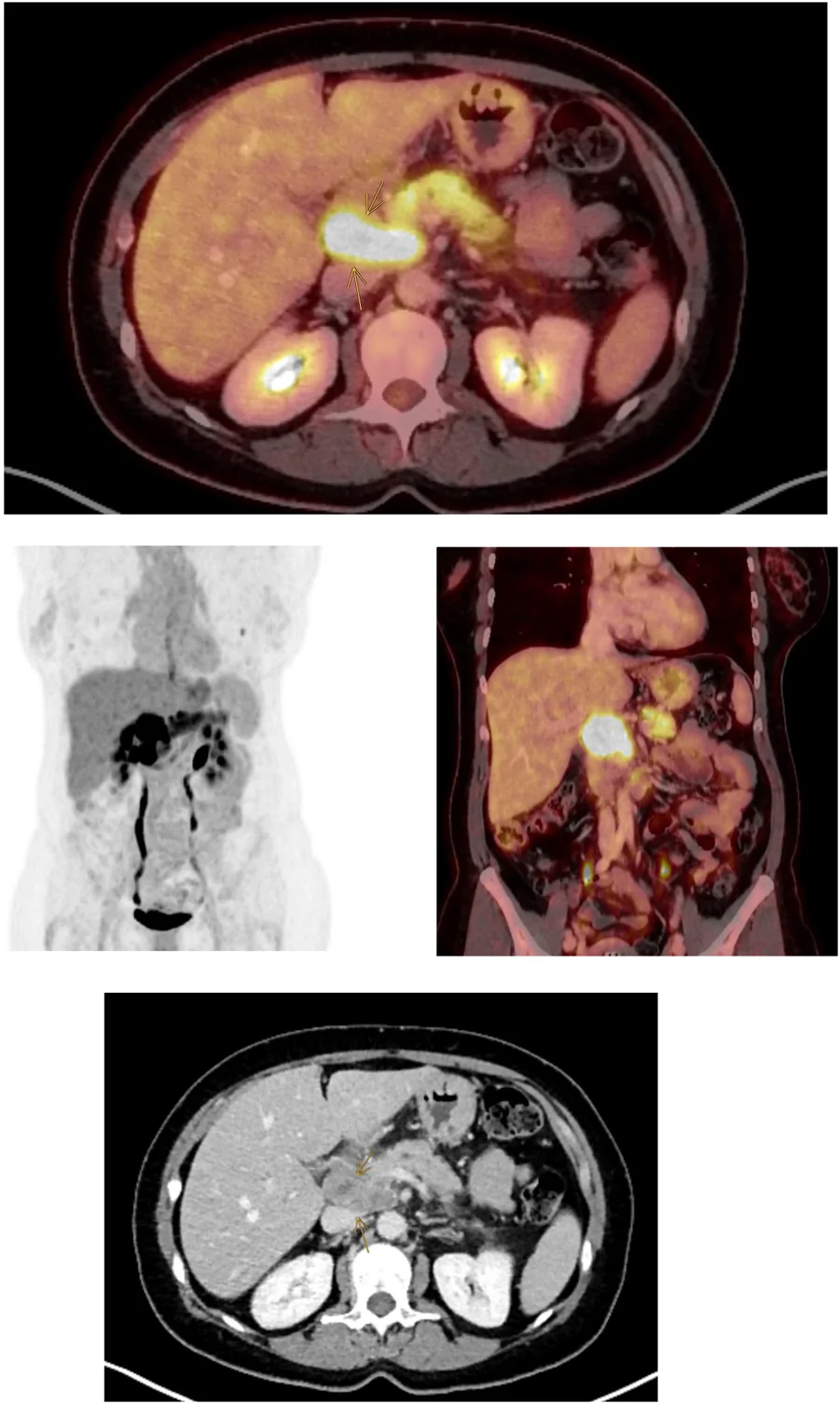
FDG-PET-CT. Large metabolically active mass measuring 2.3 × 4.8 cm between the portal vein and inferior vena cava.

Contrast-enhanced computed tomography (CT) revealed a mass located between the pancreatic head and the first portion of the duodenum. An additional lesion, suggestive of an enlarged lymph node, was identified cranial to the pancreatic head between the portal vein and inferior vena cava. Furthermore, a suspected liver metastasis in segment III and a nonspecific pulmonary nodule in the right lower lobe were noted. Subsequent ^18^F-FDG PET/CT demonstrated increased metabolic activity in the pancreaticoduodenal region (Figure 1). A large hypermetabolic mass measuring 2.3 × 4.8 cm was observed between the portal vein and inferior vena cava, along with several smaller metabolically active peripancreatic lymph nodes and a left supraclavicular lymph node. Multiple pulmonary nodules in both lungs, as well as a liver lesion in segment III, also showed increased tracer uptake.

Magnetic resonance imaging (MRI) of the liver confirmed a highly suspicious lesion in segment II and identified three additional small lesions in segments VII (2 mm), IVb (6 mm), and II (4 mm), consistent with metastatic disease.

Esophagogastroduodenoscopy revealed no significant abnormalities. Endoscopic ultrasonography demonstrated a hypoechoic lesion in the pancreatic head measuring at least 36 mm, as well as a lymph node measuring 40.5 × 18 mm.

Endoscopic ultrasound-guided fine-needle aspiration (FNA) was performed on the pancreatic mass and the portocaval lymph node, and a liver biopsy was obtained from the lesion in segment III.

FNA specimens from both the pancreatic mass and the portocaval lymph node showed malignant cells consistent with a poorly differentiated carcinoma. The tumor cells formed complex cellular clusters composed of monomorphic to pleomorphic cells with predominantly medium-sized hyperchromatic nuclei, occasional prominent nucleoli, and foamy cytoplasm. Immunocytochemistry demonstrated strong CK19 positivity, membranous and cytoplasmic β-catenin expression without nuclear localization, weak diffuse synaptophysin expression, focal trypsin positivity, and negative SOX11 staining.

**FIGURE 2:**
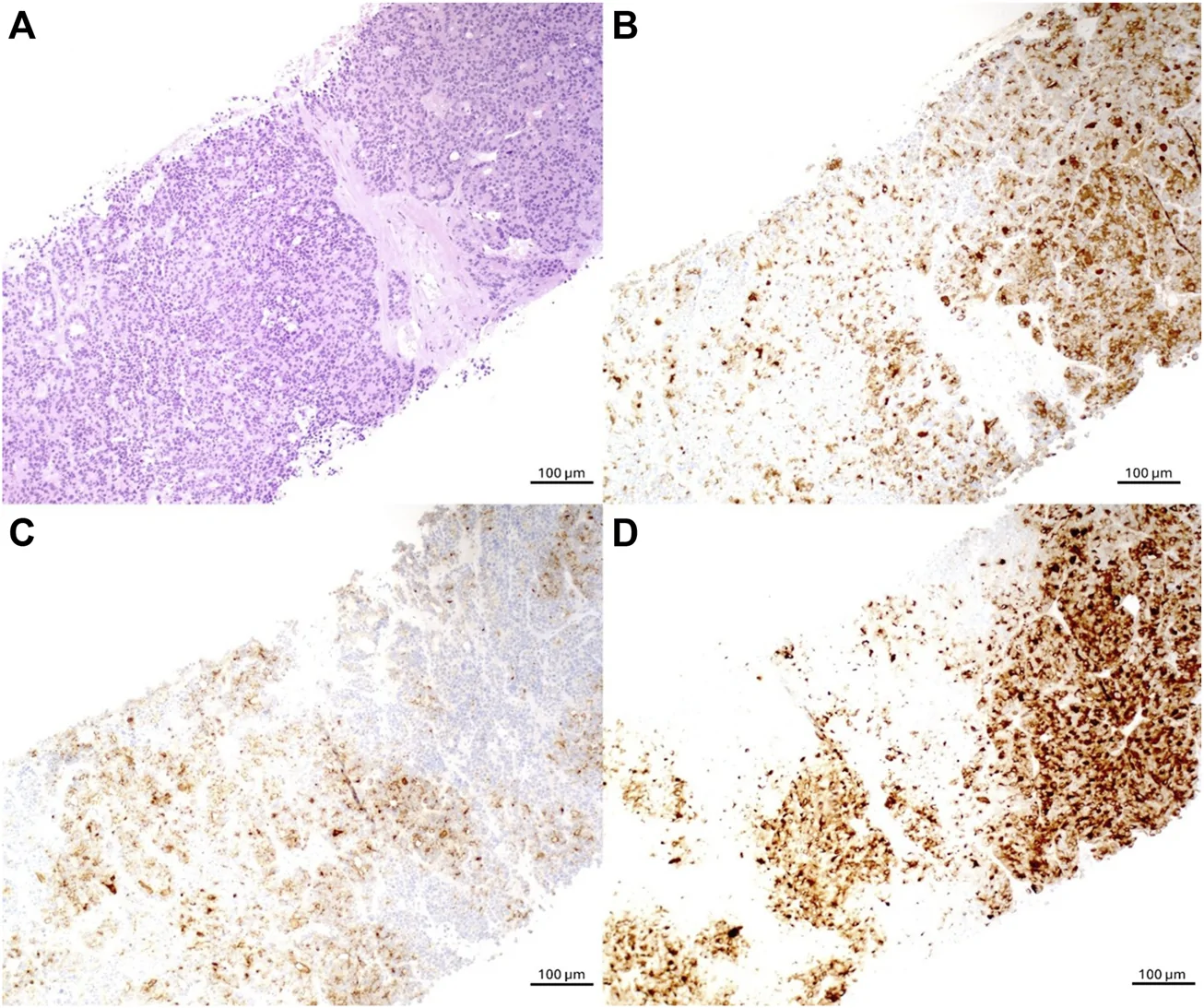
Histological and immunohistochemical characterization of pancreatic ALC with acinar differentiation. **(A)** Hematoxylin and eosin staining demonstrates a highly cellular poorly differentiated carcinoma composed of relatively uniform tumor cells separated by delicate fibrous stroma. **(B)** Trypsin immunohistochemistry demonstrates heterogeneous cytoplasmic expression, supporting acinar differentiation. **(C)** Chromogranin A immunohistochemistry shows heterogeneous neuroendocrine marker expression within the tumor. **(D)** BCL10 immunohistochemistry demonstrates cytoplasmic expression with a distribution similar to trypsin staining, further supporting acinar differentiation.

Histopathological examination revealed a solid poorly differentiated carcinoma (Figure 2A) with a high proliferative index (Ki-67 approximately 90%). Immunohistochemical analysis showed strong diffuse expression of synaptophysin, partial expression of trypsin (Figure 2B) and chromogranin A (Figure 2C), absence of CDX2 expression, loss of p53 expression, retained ATRX expression, and expression of BCL10 in tumor cells (Figure 2D). The tumour demonstrated marked mitotic activity, with up to 10 mitoses per high-power field.

Based on the initial morphological and immunohistochemical findings, including evidence of both acinar and neuroendocrine differentiation, the tumour was initially diagnosed as a mixed acinar–neuroendocrine carcinoma. However, detailed evaluation demonstrated overlapping acinar and neuroendocrine differentiation throughout the tumour, with expression of acinar markers (trypsin and BCL10) and neuroendocrine markers (synaptophysin and chromogranin A), without evidence of two spatially distinct tumour components. According to current diagnostic criteria, the tumour was therefore reclassified as pancreatic amphicrine-like carcinoma (ALC) with acinar differentiation.

Testing of predictive biomarkers by immunohistochemistry showed no evidence of mismatch repair deficiency. Comprehensive genomic profiling using next-generation sequencing (FoundationOne CDx) identified several alterations, including a RAF1 fusion, IRF2 loss, PBRM1 exon 30 loss, SMAD4 L533P, and TP53 Q52*.

Genomic analysis demonstrated microsatellite stability (MSS) and a tumour mutational burden of 5 mutations/Mb.

Based on these findings, first-line palliative chemotherapy with FOLFIRINOX (folinic acid, 5-fluorouracil, irinotecan, and oxaliplatin) was initiated, as this regimen is considered effective against both tumour components. After four cycles, the patient developed a marked elevation in liver transaminases. Endoscopic retrograde cholangiopancreatography demonstrated obstruction of the distal common bile duct caused by the pancreatic head mass, with associated dilation of the intrahepatic and extrahepatic bile ducts. A papillotomy was performed, and a covered metal stent was placed, leading to normalization of liver enzyme levels.

Restaging computed tomography after four cycles of FOLFIRINOX showed disease progression, with an increase in hepatic, pulmonary, and lymph node metastases. In light of the rapid progression, a predominance of the neuroendocrine carcinoma component was suspected. Second-line palliative chemotherapy with carboplatin and etoposide was therefore initiated. After three cycles, the primary pancreatic tumour and liver metastases remained stable; however, pulmonary and lymph node metastases continued to progress.

Due to persistent upper abdominal pain that was refractory to analgesic treatment, CT-guided percutaneous neurolysis of the coeliac plexus with alcohol was performed.

Given the presence of a RAF1 fusion and emerging evidence from published case reports, third-line palliative treatment with trametinib, a MEK inhibitor, was initiated. However, therapy had to be discontinued after 1 month because of significant treatment-related toxicity, including diarrhea, nausea, fatigue, and cutaneous adverse effects.

Following treatment discontinuation, the focus of care shifted to best supportive care.

The patient died 14 months after the initial diagnosis. No autopsy was performed.

## Discussion

Pancreatic neoplasms exhibiting combined acinar and neuroendocrine differentiation are exceptionally rare and remain diagnostically challenging. According to the current WHO Classification of Tumours (6th edition), amphicrine-like carcinoma (ALC) represents a distinct category of neoplasms characterized by combined neuroendocrine and non-neuroendocrine differentiation. These tumours should be distinguished from mixed neuroendocrine–non-neuroendocrine neoplasms (MiNENs) based on both morphology and immunophenotypic findings. MiNENs are composed of two morphologically distinct neoplastic populations, whereas ALCs demonstrate dual differentiation within the same neoplastic cells or within a diffusely admixed tumour cell population.

In the present case, the tumour showed diffuse expression of the acinar markers trypsin and BCL10 together with the neuroendocrine markers synaptophysin and INSM1 within the same neoplastic cell population, without evidence of a separate neuroendocrine component. The overlapping distribution of acinar and neuroendocrine differentiation supports classification as a pancreatic ALC with acinar differentiation rather than a MiNEN composed of distinct acinar and neuroendocrine components. This case highlights the importance of integrating histomorphology with immunohistochemistry for the accurate classification of rare pancreatic neoplasms exhibiting mixed differentiation.

Pancreatic acinar cell carcinomas (ACC) have a molecular profile distinct from pancreatic ductal adenocarcinoma and frequently harbor alterations involving the MAPK signaling pathway, including recurrent rearrangements of RAF1, BRAF, and RET [7–9]. Comprehensive genomic profiling in our patient identified a rare KANK4::RAF1 fusion together with pathogenic alterations in TP53, SMAD4, PBRM1, and IRF2. Among these findings, the KANK4::RAF1 fusion represents the principal molecular feature of this case.

RAF1 rearrangements have been described in a subset of pancreatic ACC and are considered recurrent genomic events in this tumor type [8, 9]. These rearrangements are thought to promote constitutive activation of the MAPK signaling pathway through disruption of normal RAF1 regulatory mechanisms while preserving the C-terminal RAF1 kinase domain. In our case, the identified KANK4::RAF1 fusion was predicted to retain the RAF1 kinase domain, providing a biologically plausible mechanism for activation of RAF1-dependent downstream signaling. Although KANK4::RAF1 has been reported only rarely [9], a literature search and review of available fusion databases did not identify previous reports of this fusion in pancreatic ALC with acinar differentiation. Therefore, our findings expand the molecular spectrum of this exceptionally uncommon pancreatic neoplasm.

The identification of a RAF1 fusion also has potential therapeutic implications. RAF1 fusion proteins may activate downstream MAPK signaling through RAF–MEK–ERK pathway stimulation, providing a biological rationale for therapeutic inhibition of this pathway. Based on this molecular finding, treatment with the MEK inhibitor trametinib was initiated in our patient. However, therapy was discontinued after only several weeks because of treatment-related toxicity, and the short duration of exposure precluded meaningful assessment of treatment efficacy. Therefore, this case neither supports nor refutes the effectiveness of MEK inhibition in RAF1-rearranged pancreatic neoplasms. Nevertheless, together with emerging reports of responses to MAPK pathway inhibitors in selected pancreatic acinar carcinomas, this case illustrates the potential clinical relevance of identifying actionable genomic alterations in rare pancreatic tumors [10, 11].

Additional pathogenic alterations involving TP53, SMAD4, PBRM1, and IRF2 were also detected. Although alterations in these genes have been implicated in tumor progression, chromatin remodeling, and immune regulation in various malignancies, their specific contribution to the biology of pancreatic ALC with acinar differentiation remains uncertain [12–15]. Given the single-case nature of this report, no conclusions regarding their biological significance, prognostic relevance, or potential impact on treatment response can be drawn.

In conclusion, this case highlights the diagnostic complexity of pancreatic ALC with acinar differentiation and emphasizes the importance of integrating morphology, immunohistochemistry, and comprehensive molecular profiling for accurate classification. The identification of a rare KANK4::RAF1 fusion broadens the molecular landscape of these exceptionally uncommon tumours and supports consideration of comprehensive genomic profiling in the diagnostic evaluation of rare pancreatic a neoplasms. Such profiling may reveal potentially actionable molecular alterations even in tumour entities for which established targeted treatment strategies are currently lacking [16–19].

## Data Availability

The original contributions presented in the study are included in the article/supplementary material, further inquiries can be directed to the corresponding author.
